# Automated histological classification for digital pathology images of colonoscopy specimen via deep learning

**DOI:** 10.1038/s41598-022-16885-x

**Published:** 2022-07-27

**Authors:** Sun-ju Byeon, Jungkap Park, Yoon Ah Cho, Bum-Joo Cho

**Affiliations:** 1 Department of Pathology, Hallym University Dongtan Sacred Heart Hospital, Hallym University College of Medicine, Hwaseong, Republic of Korea; 2 Medical Artificial Intelligence Center, Hallym University Sacred Heart Hospital, Anyang, Republic of Korea; 3 Department of Pathology, Hallym University Sacred Heart Hospital, Hallym University College of Medicine, Anyang, Republic of Korea; 4 Department of Ophthalmology, Hallym University Sacred Heart Hospital, Hallym University College of Medicine, 22, Gwanpyeong-ro 170 beon-gil, Dongan-gu, Anyang, Gyeonggi-do 14068 Republic of Korea

**Keywords:** Machine learning, Gastrointestinal diseases, Colonoscopy, Pathology

## Abstract

Colonoscopy is an effective tool to detect colorectal lesions and needs the support of pathological diagnosis. This study aimed to develop and validate deep learning models that automatically classify digital pathology images of colon lesions obtained from colonoscopy-related specimen. Histopathological slides of colonoscopic biopsy or resection specimens were collected and grouped into six classes by disease category: adenocarcinoma, tubular adenoma (TA), traditional serrated adenoma (TSA), sessile serrated adenoma (SSA), hyperplastic polyp (HP), and non-specific lesions. Digital photographs were taken of each pathological slide to fine-tune two pre-trained convolutional neural networks, and the model performances were evaluated. A total of 1865 images were included from 703 patients, of which 10% were used as a test dataset. For six-class classification, the mean diagnostic accuracy was 97.3% (95% confidence interval [CI], 96.0–98.6%) by DenseNet-161 and 95.9% (95% CI 94.1–97.7%) by EfficientNet-B7. The per-class area under the receiver operating characteristic curve (AUC) was highest for adenocarcinoma (1.000; 95% CI 0.999–1.000) by DenseNet-161 and TSA (1.000; 95% CI 1.000–1.000) by EfficientNet-B7. The lowest per-class AUCs were still excellent: 0.991 (95% CI 0.983–0.999) for HP by DenseNet-161 and 0.995 for SSA (95% CI 0.992–0.998) by EfficientNet-B7. Deep learning models achieved excellent performances for discriminating adenocarcinoma from non-adenocarcinoma lesions with an AUC of 0.995 or 0.998. The pathognomonic area for each class was appropriately highlighted in digital images by saliency map, particularly focusing epithelial lesions. Deep learning models might be a useful tool to help the diagnosis for pathologic slides of colonoscopy-related specimens.

## Introduction

Colonoscopy is an effective tool to detect and discriminate colorectal lesions including colorectal cancer, polyps, or inflammatory bowel disease, and the number of colonoscopy examination is increasing in various countries worldwide^1^. The utility of colonoscopy is supported by reliable histopathological readings for biopsied or excised specimens, but the number of new pathologists has decreased annually (mean 41.4 in 2010–2012 vs. mean 31.7 in 2018–2020 in Korea; a decrease of 17.53% between 2007 and 2017 in the United States)^2^. This mismatch between the demand and supply for pathologists would cause workload for doctors, and may increase the chance of misdiagnosis^3,4^. Thus, a significant need has arisen for supporting pathologists.

Recently, artificial intelligence (AI) using machine learning or deep learning algorithm has been rapidly introduced in medicine and has provided automated computer-aided diagnosis to help doctors^5^. In pathology, AI researches have focused on the diagnosis and grading of diseases in digital pathology images^6^. Small metastatic tumor cells can be found with high accuracy in sentinel lymph node biopsy during breast cancer surgery, and this algorithm could reduce the labor burden of pathologists on frozen biopsy^7,8^. For colorectal polyps, deep learning model achieved the accuracy of 93.0% for lesion classification using 239 whole-slide imaging (WSI) images^9^. The performance of deep learning model highly depends on the data size and quality; the diagnostic accuracy for large intestine pathology was 92.5% when 1000 images were used, and decreased to 88% for 100 images and 59.8% for 10 images^10^.

Thus far, there have been very few deep learning studies focused on colonoscopy-related specimens. For gathering the pathological images, the WSI method has several strengths, but there are still many institutions not having the slide scanner system because of the high cost^11,12^. Therefore, in the present study, we aimed to develop and validate an efficiently performing deep learning model for colonoscopy biopsy or excision specimens using non-WSI colon pathology images, to reduce barriers in constructing the training dataset for deep learning. We trained two types of recent deep learning architectures with non-WSI digital photographs of pathology slides and validated the efficacy of the AI model in an independent test dataset.

## Materials and methods

### Data collection

Patients who underwent colonoscopy procedures, including biopsy, polypectomy, mucosal resection, and submucosal dissection, at Hallym University Dongtan Sacred Heart Hospital between 2017 and 2019 were consecutively included. To compensate for the small number of traditional serrated adenoma (TSA) cases, additional slides were included from 48 patients diagnosed with TSA who underwent endoscopic or surgical biopsy at Hallym University Sacred Heart Hospital between 2015 and 2019. All biopsy specimens were fixed in 10% neutral buffered formalin for a sufficient time (almost overnight), paraffin-embedded, sectioned into 5-µm-thick slices, and stained with hematoxylin and eosin. Two pathology laboratories were passed for the quality control program of the Korean Society of Pathology from 2015 to 2019. This research was conducted after receiving approval from the institutional review boards (IRBs) of the participating hospitals (IRB no. HDT 2020-03-009 and HALLYM 2021-10-015, respectively) and was carried out in accordance with the Declaration of Helsinki. The IRBs waived the requirement of written informed consent because the present study involved no more than the minimal risk to subjects.

From the collected slides, deterministic fields were chosen by experienced pathologists (1–3 images per lesion without overlap) using a Nikon Eclipse 80i microscope with a Nikon Plan Apo 10×/0.45 objective. To minimize any possible differences in the photography condition, the auto-exposure function of the camera was turned off and the white-balancing condition was fixed before taking photographs. Then, digital photographs were taken using the NIS Elements BR program with a DS-Ri2 camera. The light source was a 100 W lamp fixed with maximum brightness, the microscope's basic filter was used, the aperture was opened as much as possible, and the trinocular head was set to pass light through the camera and the eyepiece. The exposure time was fixed at 64 ms and the analog gain at × 2.2. The pathology images were saved in TIFF format with a resolution of 4908 × 3264 pixels.

### Dataset construction

All images were classified into six classes according to the pathologic diagnosis. The classes were determined according to the common categories in routine colorectal pathologic examination: tubular adenocarcinoma, tubular adenoma with low-grade dysplasia (TA), TSA with low-grade dysplasia, sessile serrated adenoma (SSA) with no dysplasia, hyperplastic polyp (HP), and non-specific change (NC)^13^. The subtypes of colonoscopy specimen have been distinguished by the morphology and staining pattern of the cells and the tissues (Supplementary Fig. S1). For example, HPs are characterized by superficial serrated epithelium and funnel-shaped evenly spaced crypts with proliferative zones confined to the crypt base. The crypts do not show basal dilatation, substantial distortion, or submucosal misplacement. SSA has bland cytology and crypts with prominent serrations^13^. The distinguishing feature of SSA is an overall distortion of the normal crypt architecture, probably resulting from alternations of the proliferative zone. The most distinctive features of TSA are the slit-like serration, reminiscent of the narrow slits in the normal small intestinal mucosa, and the tall columnar cells with intensely eosinophilic cytoplasm and pencillate nuclei. In tubular adenomas, the normal crypt architecture is largely conserved, with variable elongation of the crypts and an increase in the number of glands. The epithelium shows enlarged, hyperchromatic nuclei, with varying degrees of nuclear spindling and stratification and with loss of polarity. In colorectal adenocarcinoma, the defining feature is invasion through the muscularis mucosae into the submucosa. The tumor is composed of dilated or slit-like branching tubules of variable diameter. Additionally, all images were classified into two groups from a different point of view to build a deep learning model discriminating malignant from non-malignant lesions: the adenocarcinoma group vs. the non-adenocarcinoma group. The non-adenocarcinoma group included the TA, TSA, SSA, HP, and NC classes other than adenocarcinoma.

After labeling the images, all images were resized into a 613 × 408 pixels format as small as one eighth of the original resolution, to reduce the image resolution uniformly, and thus to enable the GPU memory to deal with the data size and increase the learning speed of deep learning models. A patch extraction was not adopted. Before training deep learning models, the images were normalized using the means (0.485, 0.456, and 0.406 for the red, green, and blue channels) and standard deviations (0.229, 0.224, and 0.225 for the red, green, and blue channels) of the ImageNet dataset. Image histogram equalization or data augmentation using horizontal or vertical flipping was not applied, because these did not significantly improve the model performances in our pilot study.

Then, the entire dataset was divided into a training dataset, a tuning dataset, and a test dataset at a ratio of 8:1:1, three times. Each dataset split was performed using the anonymized patients’ ID as a key to prevent one lesion of a certain patient from belonging to both the training and the test datasets. The dataset split was performed three times, and the average performance was evaluated.

### Deep learning model training

Two convolutional neural network (CNN) architectures were adopted which showed the better performances in our pilot studies: DenseNet-161 (https://pytorch.org/hub/pytorch_vision_densenet; accessed on 3 June 2021) and EfficientNet-B7 (https://github.com/lukemelas/EfficientNet-PyTorch; accessed on 3 June 2021). The details of these architectures are described elsewhere^14,15^. Briefly, the DenseNet architecture involves dense blocks that give the concatenated input feature maps of several former sub-blocks to the next sub-block as the input feature^14^. Meanwhile, the EfficientNet-B7 architecture belongs to one of the state-of-the-art algorithms and contains an MBconv block balanced for the width and depth of the architecture by reinforcement learning^15^. These architectures were pre-trained using the ImageNet dataset, and the pre-trained models were fine-tuned using the training dataset in this study. All parameters of the convolutional and fully-connected layers were unfrozen and fine-tuned.

The input image size was 613 × 408 pixels. Categorical cross-entropy was used as a loss function, and the Adam optimizer with a β1 = 0.9 and a β2 = 0.999 was adopted. The batch size was 15 for DenseNet-161 and 8 for EfficientNet-B7. The initial learning rate was 1e-4, and a learning rate decay was not used. The number of training epochs was 100. The point that minimized the validation loss in the tuning set was selected. The CNN models were implemented on the PyTorch platform and trained using a customized water-cooling system deep learning server equipped with NVIDIA GeForce Titan RTX 4-way graphics processing units.

To implement a saliency map presenting the region used as the basis for decision, gradient-weighted class activation mapping (Grad-CAM) was applied^16^. The region of interest of the deep learning model was presented as a color map, and the Grad-CAM images of the test dataset were reviewed by an experienced human pathologist (S.J.B).

### Main outcome measures and statistical analyses

To evaluate the model performance, the mean accuracies of the models were calculated three times using different test datasets. The mean sensitivity, specificity, positive predictive value, and negative predictive value were also evaluated. Receiver operating characteristic (ROC) curves and the area under the ROC curve (AUC) were obtained. Continuous variables are presented as means and 95% confidence intervals (CIs).

## Results

Ultimately, 1865 digital pathological images were included from 703 patients in the study. The composition of the training dataset, tuning dataset, and test dataset of the first data split are presented in Table 1. The majority class was adenocarcinoma (429 images [23.0%]), and the minority class was TSA (150 images [8.0%]).

**Table 1:** Data composition for the firstly split datasets for building deep learning models.

	Whole dataset		Training set		Tuning set		Test set	
	Image N	Patient N	Image N	Patient N	Image N	Patient N	Image N	Patient N
Overall	1865	703	1484	561	173	71	208	71
ADC	429	206	332	158	42	23	55	25
TA	462	231	357	179	46	25	59	27
TSA	150	71	137	61	3	3	10	7
SSA	278	192	220	149	34	26	24	17
HP	189	161	154	132	12	10	23	19
NC	357	261	284	208	36	28	37	25

*ADC* advanced tubular adenocarcinoma, *TA* tubular adenoma, *TSA* traditional serrated adenoma, *SSA* sessile serrated adenoma, *HP* hyperplastic polyp, *NC* nonspecific change.

### Multi-class classification

**Figure 1:**
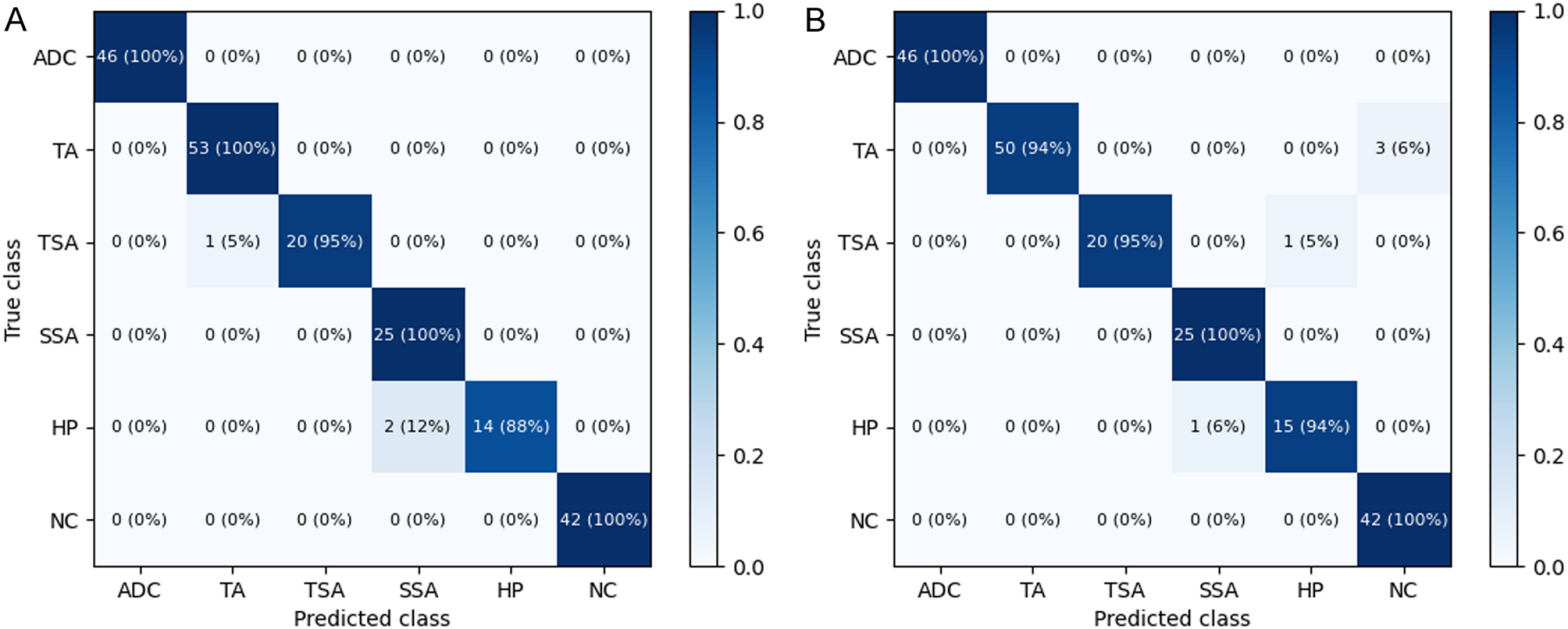
Heatmap of confusion matrix for the best-performing models: (**A**) DenseNet-161 (**B**) EfficientNet-B7.

**Figure 2:**
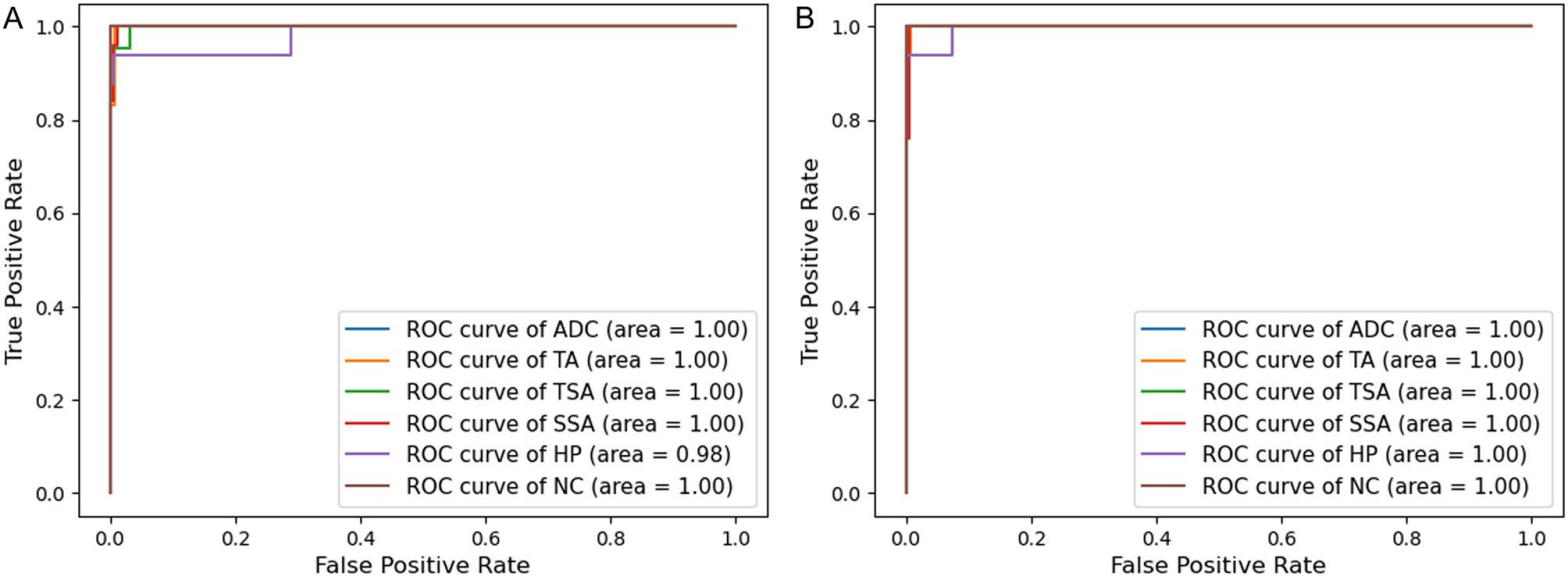
Per-class receiver operating characteristic curves for the best-performing models: (**A**) DenseNet-161 (**B**) EfficientNet-B7.

The mean accuracy for classifying pathologic images into six classes was 97.3% (95% CI 96.0–98.6%) by DenseNet-161 and 95.9% (95% CI 94.1–97.7%) by EfficientNet-B7. The classification confusion matrices of the best-performing models for each architecture are shown in Fig. 1. The per-class performances are presented in Table 2. Both deep learning models showed very high sensitivity and specificity values of over 95% for all six classes. The mean per-class AUC was highest in the adenocarcinoma class (1.000; 95% CI 0.999–1.000) by DenseNet-161 and in the TSA class (1.000; 95% CI 1.000–1.000) by EfficientNet-B7. The mean per-class AUC was lowest in the HP group (0.991; 95% CI 0.983–0.999) by DenseNet-161 and in the SSA group (0.995; 95% CI 0.992–0.998) by EfficientNet-B7, but the AUC value was still over 0.99. The per-class ROC curves of the best-performing models for each architecture are shown in Fig. 2.

**Table 2:** Per-class model performances of deep learning models for six-class classification.

Model	Sensitivity (%)	Specificity (%)	PPV (%)	NPV (%)	F1 score (%)	AUC (95% CI)
**DenseNet-161**						
ADC	99.4 (98.4–100.0)	99.6 (98.9–100.0)	98.2 (95.4–100.0)	99.8 (99.4–100.0)	98.8 (97.5–100.0)	1.000 (0.999–1.000)
TA	97.7 (95.3–100.0)	98.7 (97.6–99.7)	95.6 (91.3–99.8)	99.3 (98.7–99.9)	96.6 (93.3–99.9)	0.995 (0.989–1.000)
TSA	98.4 (95.9–100.0)	100.0 (100.0–100.0)	100.0 (100.0–100.0)	99.8 (99.5–100.0)	99.2 (97.9–100.0)	0.999 (0.999–1.000)
SSA	96.5 (93.4–99.5)	99.1 (98.8–99.3)	93.8 (91.4–96.1)	99.4 (98.8–100.0)	95.0 (94.0–96.1)	0.993 (0.985–1.000)
HP	91.5 (87.7–95.3)	99.6 (99.3–99.9)	97.0 (94.7–99.4)	99.1 (98.8–99.3)	94.1 (92.9–95.3)	0.991 (0.983–0.999)
NC	97.7 (94.0–100.0)	99.8 (99.5–100.0)	99.1 (97.7–100.0)	99.3 (98.3–100.0)	98.4 (96.7–100.0)	0.995 (0.986–1.000)
**EfficientNet-B7**						
ADC	97.3 (94.7–99.8)	99.8 (99.4–100.0)	99.1 (97.6–100.0)	99.1 (98.2–100.0)	98.1 (96.7–99.6)	0.997 (0.991–1.000)
TA	95.3 (94.2–96.4)	97.8 (95.6–100.0)	93.9 (88.7–99.0)	98.4 (98.1–98.8)	94.5 (92.3–96.6)	0.997 (0.994–0.999)
TSA	95.1 (90.5–99.7)	100.0 (100.0–100.0)	100.0 (100.0–100.0)	99.7 (99.4–99.9)	97.4 (95.0–99.9)	1.000 (1.000–1.000)
SSA	97.5 (95.5–99.6)	99.2 (99.0–99.5)	95.0 (92.6–97.4)	99.6 (99.3–99.9)	96.2 (94.3–98.2)	0.995 (0.992–0.998)
HP	93.6 (87.5–99.6)	99.2 (98.9–99.6)	93.7 (92.2–95.2)	99.3 (98.5–100.0)	93.5 (91.2–95.8)	0.995 (0.995–0.995)
NC	95.0 (90.9–99.0)	98.8 (97.8–99.8)	95.1 (91.1–99.1)	98.8 (97.7–99.8)	94.9 (92.5–97.4)	0.997 (0.994–1.000)

*PPV* positive predictive value, *NPV* negative predictive value, *AUC* area under the receiver operating characteristic curve, *CI* confidence interval, *ADC* advanced tubular adenocarcinoma, *TA* tubular adenoma, *TSA* traditional serrated adenoma, *SSA* sessile serrated adenoma, *HP* hyperplastic polyp, *NC* nonspecific change.

### Binary classification

For binary classification discriminating the adenocarcinoma group from the non-adenocarcinoma group, the mean accuracy was 99.2% (95% CI 98.9–99.4%) by DenseNet-161 and 99.5% (95% CI 99.0–100.0%) by EfficientNet-B7. The performances of the two architectures are presented in Table 3. The mean AUC was 0.995 for DenseNet-161 and 0.998 for EfficientNet-B7. The sensitivity level was 97.1% by DenseNet-161 and 98.5% by EfficientNet-B7 at a specificity level of 99.8%.

**Table 3:** Model performances of deep learning models for binary classification discriminating advanced colorectal adenocarcinoma.

Model	Sensitivity (%)	Specificity (%)	PPV (%)	NPV (%)	F1 score (%)	AUC (95% CI)
DenseNet-161	97.1 (96.5–97.8)	99.8 (99.4–100.0)	99.1 (97.6–100.0)	99.1 (98.8–99.5)	98.1 (97.3–98.9)	0.995 (0.988–1.000)
EfficientNet-B7	98.5 (97.2–99.8)	99.8 (99.4–100.0)	99.1 (97.6–100.0)	99.6 (99.2–99.9)	98.8 (97.5–100.0)	0.998 (0.995–1.000)

*PPV* positive predictive value, *NPV* negative predictive value, *AUC* area under the receiver operating characteristic curve, *CI* confidence interval.

### Grad-CAM

**Figure 3:**
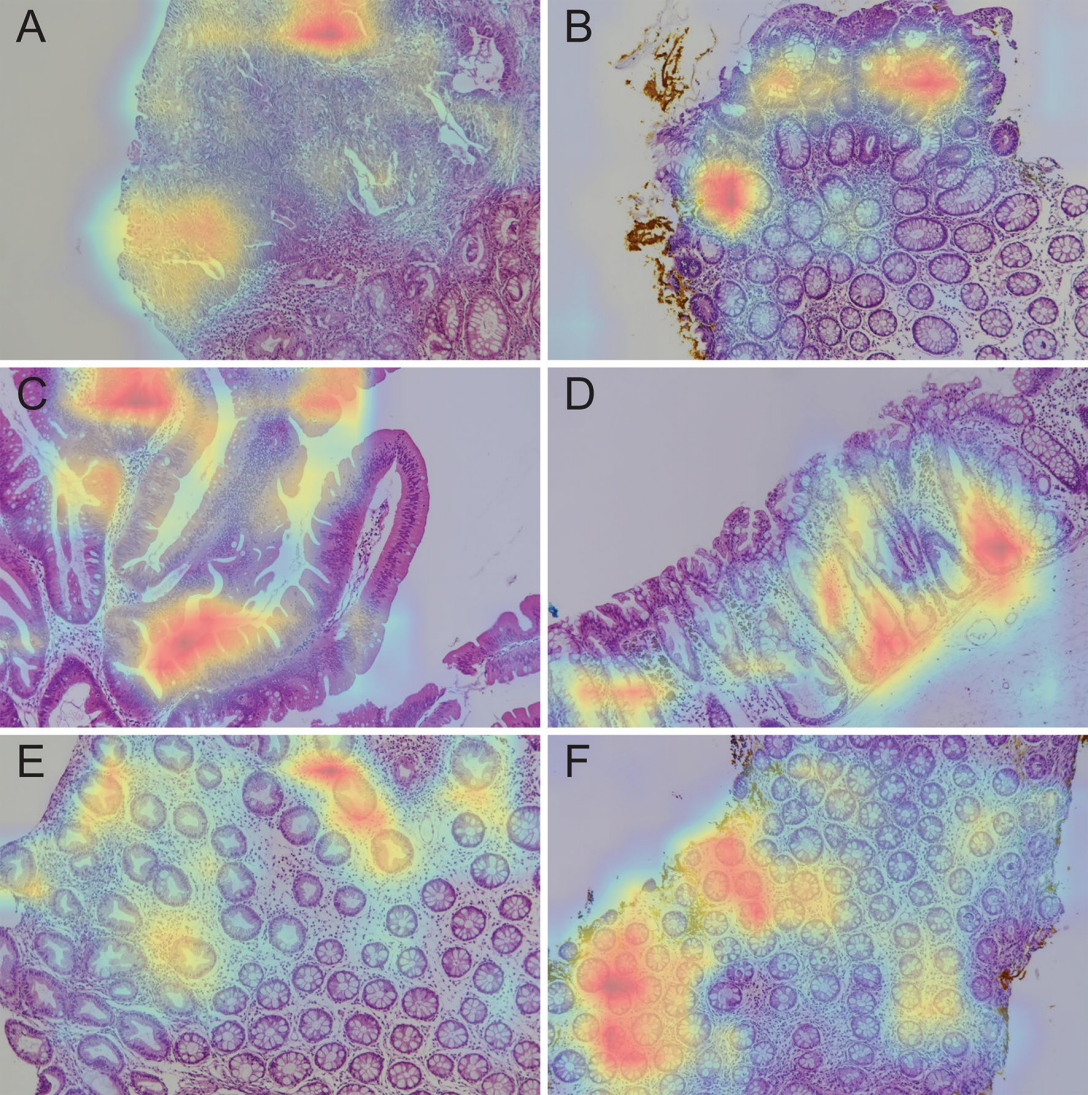
Representative images of Grad-CAM for each class: (**A**) adenocarcinoma, (**B**) tubular adenoma, (**C**) traditional serrated adenoma, (**D**) sessile serrated adenoma, (**E**) hyperplastic polyp, and (**F**) non-specific change.

Representative Grad-CAM images for determining each class in the test dataset are shown in Fig. 3. In most cases, we confirmed that deep learning models classify each class by assigning weights to epithelial lesions appropriately. In most cases, not all actual pathognomonic areas were weighted, but the highlighted areas coincided with the epithelial pathognomonic area of the lesions.

## Discussion

In this study, deep learning models showed very high level of accuracy in discriminating six histopathological classes of colon lesion, without region-based annotation. Non-WSI camera-based images achieved sufficient classification performance exceeding 95% for the accuracy. Although the histological region is a complex mixture of epithelial lesion, surrounding normal epithelial component, and additional mesenchymal component, the Grad-CAM focused on the appropriate key areas of the epithelial lesions, not on other irrelevant surrounding areas.

Recently, there have been several studies predicting the histological type of lesions on colonoscopy images using AI^17,18^. Nevertheless, fewer studies have investigated the pathologic diagnosis of the histologic slide images of colon polyps compared to endoscopic imaging studies. Most colon pathology AI studies have aimed to distinguish adenocarcinoma from non-adenocarcinoma^10,19–21^. Iizuka et al. classified colon pathology images using AI, including normal, adenoma (probably mainly TA), and adenocarcinoma^22^. When classified using a recurrent neural network, the AUC of TA and adenocarcinoma was 0.964 and 0.975, respectively, slightly lower than the results of our study. Compared with the research of Iizuka et al., our research has the following differences: They extracted the lesion image using random sampling, but in our study, the pathology image was taken by a pathologist who selected a representative lesion. Their study used Inception-v3 was used as the AI model, whereas ours used DenseNet-161 and EfficientNet-B7. The number of images used to train the model also differed, but the extent to which this affects the model accuracy is unknown.

According to Jones et al., when AI was trained using 50 and 30 unambiguous benign and malignant images of the colon, the accuracy was 92.3% and 82.5%, respectively^10^. In the case of a malignant tumor (especially adenocarcinoma) of the colon, it is relatively easy to distinguish it from other lesions by pathologic findings due to the characteristics of cytologic atypia, disruptive and invasive growth of surrounding tissues, and desmoplastic stroma. Therefore, even with only 30–50 images, we believe that adenocarcinoma can be easily learned using AI with an accuracy of 90%. In our study, although the dataset was reduced to 1/4, the adenocarcinoma was 100% predictable. SSA, TA, and TSA, which are predicted to be morphologically easy to recognize patterns, showed an accuracy of more than 90% even with use of a 1/4 dataset. In our study, the 1/4 data set used an average of 69 images for each disease group, and the accuracy was almost 90% after the data augmentation process. For lesions with subtle pathological changes (e.g., collagenous colitis or amyloid colitis), we cannot predict the amount of learning data needed to ensure sufficient accuracy for diagnosis^23^. However, we believe that if researchers could collect typical cases and use them for learning, they will be able to build an AI model with sufficient accuracy using fewer cases.

Grad-CAM is a method that allows researchers to easily identify the areas to which weights are assigned in classification using a CNN. Therefore, if Grad-CAM assigns weights to appropriate parts, it will be a useful model. Otherwise, an overall re-evaluation is necessary throughout the model learning process. In our pilot study, we were able to confirm through Grad-CAM that the part that contributed significantly to the determination of SSA was the empty space in the submucosal area of the endoscopic submucosal dissection specimen. We added an image processing step so that areas without cells in certain regions were omitted from the learning process, allowing the AI algorithm to focus more on the colon’s epithelial layer. We evaluated our model using Grad-CAM images for all datasets using the trained model. In most cases, our model weights epithelial components, not the stroma. This indicates that if most colon polyps are epithelial lesions, the widely used CNN model works well, even when applied to the pathologic classification of colon polyps. The classes of the image classification CNN model are independent of each other. However, pathological lesions can only be present in a small part of the entire image. In this case, an error occurs when classifying the image as an NC instead of as a lesion. To solve this problem, weight correction according to class can be easily applied. The appropriate correction value requires further study. HP and SSA have common microscopic findings showing serration protruding the crypt lumen. However, unlike HP, SSA has base crypt dilatation. If the muscularis mucosa is always visible, this may affect the CNN model training process. To effectively learn SSA in the CNN model, a new concept that considers the direction of the image in the model training process is required. In the case of adenocarcinoma, solid growth (undifferentiated carcinoma-like area) and signet-ring cell components had lighter weights. These tumors are rare, but they have a worse prognosis; therefore, so we think it is important to be reflected in the diagnosis process. We also judge that it would be better to add additional cases and assign weights as separate classes rather than as adenocarcinoma classes. In this study, we used a method in which the CNN model focused more on the epithelium by removing the large submucosa area and the blank space of the slide by zero-filling. However, to learn epithelial lesions more effectively, a technique that removes a smaller empty space and stroma is needed.

Among the datasets used in the AI algorithm learning process, several images were used from several polyps removed from one patient. In general, if the dataset is not large enough, the use of multiple data originating from one source has the potential to distort the learning outcome. However, it is common to use multiple images (WSI segmentation) from one source (one patient) in pathologic image research. This is because even a single lesion is morphologically or genetically heterogeneous. Malignant tumors are defined as tumors originating from one cell and clonal expansion in which sufficient genetic mutations are accumulated to enable uncontrolled cell division. However, the adjacent tumor cells are not completely identical because they exhibit genetic intra-tumoral heterogeneity through clonal evolution, in which several genetic abnormalities independently occur during the proliferation process^24^. The colon adenomatous polyp also showed intra-tumoral heterogeneity examined by a single-cell sequencing technique^25,26^. These studies showed that even non-proliferative and healthy colon epithelia are not identical at the cellular level^27,28^. Therefore, it is reasonable to assume that all images are independent if they do not overlap with each other.

In this study, typical images determined by a pathologist were used as the dataset. For the pathology image to be “typical,” the lesion should be large enough for a diagnosis. However, adenocarcinoma should be diagnosed even if it is small in size, with only a few cells or a few glands. Technically, the CNN-based AI algorithm can detect diminutive adenocarcinomas with sufficient training data. It is not known how much data are needed to detect individual scattered adenocarcinoma cells using the AI algorithm. Since it was not our study goal to create an AI model that can judge very diminutive lesions, there is the limitation that we could not detect subtle changes in this study.

One of the main limitation of the current study is the use of individual tiles instead of the WSI images. The lack of the slide scanner system in our institution was the reason, and working on individual tiles instead of WSIs would decrease the utility value of automated deep learning models. In the future, researches using WSI images obtained by an automated scanner would be much more helpful. Another limitation of this study is the absence of technical novelty for the CNN structures: we adopted off-the-shelf CNN structures open to the public. Nevertheless, this study could be said to be an early study that showed that deep learning has good performance even on the pathology of colonoscopy specimens. Also, to validate the reliability of the model performances more clearly, additional validation on an external dataset would be needed in the future. In addition, to show the effectiveness or necessity of deep learning models on this task, a comparative study with a radiomics-based method having a much lighter model would be also required in the future researches. The lack of comparison with traditional machine learning methods was another limitation of the present study. This was because we could not find an established technique that can be easily implemented for 3-channel images. Comparative researches would show the difference in the performances among different methods in the future.

In conclusion, this study showed that deep learning models classify histopathologic images with high accuracy into six types of commonly encountered diagnoses in colonoscopy specimens using relatively less data. Pathologists could be assisted by deep learning models also in diagnosing the colonoscopy-related specimens. Future researches would be required to build a more comprehensive deep learning model diagnosing more various types of colorectal lesion.

## Supplementary Information

Supplementary Figure S1.

## Data Availability

The datasets used and/or analyzed during the current study are available from the corresponding author on reasonable request, if approved by the institutional review boards of the participating hospitals.
