# Supplementary material for: Automated histological classification for digital pathology images of colonoscopy specimen via deep learning
Source: Sci Rep. 2022 Jul 27;12:12804. doi: 10.1038/s41598-022-16885-x (PMC9329279; doi:10.1038/s41598-022-16885-x)

**Supplementary Figure S1.** Representative images for histopathological subtypes of colonoscopy specimen: (A) hyperplastic polyp, (B) sessile serrated adenoma, (C) traditional serrated adenoma, (D) tubular adenoma, (E) adenocarcinoma, and (F) non-specific change.


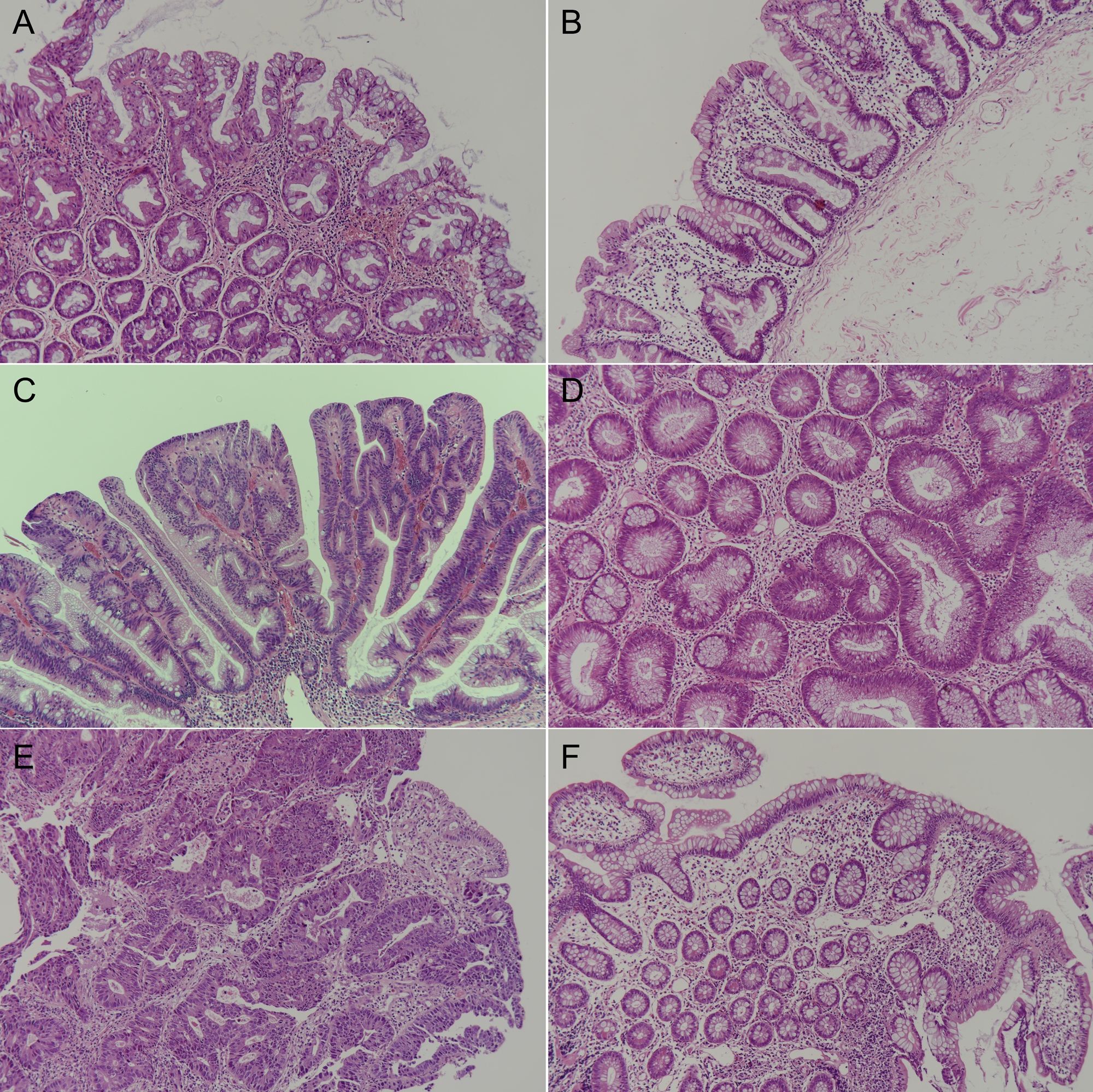

Supplement: Supplementary file 1 — Supplementary Figure S1. [file 41598_2022_16885_MOESM1_ESM.docx]
